# Heterogeneity and Plasticity of Human Breast Cancer Cells in Response to Molecularly-Targeted Drugs

**DOI:** 10.3389/fonc.2019.01070

**Published:** 2019-10-15

**Authors:** Emira Bousoik, Ramina Nabiee, Farideh Amirrad, Ashley Nichols, Rebecca Witt, Parvin Mahdipoor, Hamidreza Montazeri Aliabadi

**Affiliations:** Department of Biomedical and Pharmaceutical Sciences, Chapman University School of Pharmacy, Harry and Diane Rinker Health Science Campus, Irvine, CA, United States

**Keywords:** breast cancer, resistance, plasticity, heterogeneity, molecularly-targeted drugs

## Abstract

Non-responsive subpopulation of tumor cells, and acquired resistance in initially responsive cells are major challenges for cancer therapy with molecularly-targeted drugs. While point mutations are considered the major contributing factor to acquired resistance, in this study we explored the role of heterogeneity and plasticity of selected human breast cancer cell lines (MDA-MB-231, MDA-MB-468, and AU565) in their initial and adjusted response, respectively, to ruxolitinib, everolimus, and erlotinib. After determination of lethal concentration for 50% cell death (LC50), cells were exposed to selected drugs using three different approaches: single exposure to 4 × LC50 and collection of surviving cells, multiple exposures to 1.5 × LC50 and monitoring the surviving population, and exposure to gradually increasing concentrations of selected drugs (range of concentrations equivalent to 10% of LC50 to 1.5 × LC50). Surviving cells were studied for adjustments in expression level of selected proteins using quantitative PCR and Western Blot. Our data indicated overexpression of a variety of proteins in resistant populations, which included cell membrane receptors EGFR and HER2, anti-apoptotic proteins Bcl-2 and BIRC8, and other proteins involved in cell signaling (e.g., Akt1, MAPK7, and RPS6KA5). Silencing the identified alternative proteins via siRNA resulted in significant drop in the LC50 of the selected molecularly-targeted drugs cells resistant to ruxolitinib (via targeting Akt), everolimus (via targeting EGFR, MAPK7, RPS6KA5, and HER2), and erlotinib (via silencing Bcl2 and BIRC8). Our data indicates that targeting well-selected alternative proteins could potentially sensitize the resistant cells to the effect of the molecularly-targeted treatment.

## Introduction

Despite usually promising initial response to newly developed molecularly-targeted drugs (used individually or in combinations) (1), an initial unresponsive sub-population (force-selected by the drug) and development of acquired resistance after repeated exposures seem to be inevitable in cancer cells (2). The innate resistance is usually explained by tumor heterogeneity. Several hypotheses have been proposed to explain the well-documented intra- and inter-population heterogeneity of cancer cells. A recent genomic profiling study in 349 patients with colorectal tumors suggests that this type of tumors grow predominantly as a single expansion populated by numerous intermixed sub-clones, and verifies that most detectable intra-tumor heterogeneity occurs early during tumor growth and leads to spatial heterogeneity in each tumor (3). More diverse pattern has been reported in other types of cancer, including acute lymphoblastic leukemia (4). While majority of the cells in a given tumor might respond to the molecularly-targeted drugs, a sub-population usually survives the treatment, which results in a “Darwinian clone selection” and tumor relapse (5). On the other hand, tumor cells could also “acquire” resistance as a result of incomplete response and multiple exposures to the same anticancer drug. While multiple mechanisms are involved in this type of resistance (among which point mutations in the gene expressing the targeted protein is well-accepted), plasticity of cancer cells could play a crucial role. While cells rely on an “addiction pathway” (specific overexpressed and/or overactivated pathway), exposure to molecularly-targeted drugs could prompt the cells to bypath the targeted protein by activating alternative or “off-target” proteins and/or pathways (6).

Multiple pathways play an oncogenic role in different cancer types, and JAK/STAT, PI3K/Akt, and Ras/Raf/MEK/ERK pathways are major pathways that have been studied extensively. JAK/STAT pathway is associated with a variety of transmembrane receptors. JAK overactivation has been implicated in tumorigenesis (7), and persistent activation of STAT3, and to a lesser extent STAT5, has been shown to increase proliferation, survival, angiogenesis, and metastasis in a variety of human cancers (8). After transportation into nucleus, STAT3 acts as a transcription factor, and enhances expression of antiapoptotic proteins [including Mcl-1 (9), Bcl-2 (10), and surviving (11)], proteins involved in cell-cycle progression [including c-Myc and cyclin D1 (12)], and angiogenesis [including Vascular Endothelial Growth Factor, VEGF (13)]. We have recently reviewed the crucial role of JAK/STAT pathway in tumorigenic cells (14). PI3K/AKT pathway is one of the most commonly disrupted pathways in cancer, and the PI3K-dependent activation of the serine/threonine kinase AKT is a key factor in many survival mechanisms (15). The main downstream effector of PI3K/Akt pathway is mechanistic target of rapamycin (mTOR) (16). Binding of growth factors to cell surface receptors could also activate Ras/Raf/MEK/ERK pathway. Mutations in KRAS, BRAF, MEK1, or MEK2 result in growth factor-independent ERK1 and ERK2 activation, which enhances cell proliferation and survival (17). While proteins involved in these mechanisms are traditionally categorized in specific “pathways,” extensive “crosstalk” among pathways has been also reported. Activation of mTOR downstream effectors by p38 MAPK pathway (18), the effect of STAT3 activation on Ras and PI3K/Akt pathway (19), and JAK2 activation on PI3K and ERK pathways (20, 21) are examples of these crosstalks, which could be an important factor in the ability of cancer cells to “switch” to an alternative pathway for survival.

In this study, we hypothesize that human breast cancer cell lines could demonstrate a similar heterogeneity and plasticity in exposure to molecularly-targeted drugs, and we aim to study the alternative proteins that potentially play a role in inherent and acquired resistance to these anticancer agents. We selected three breast cancer cell lines, and three molecularly-targeted drugs: (a) Ruxolitinib (RUX; pan-JAK inhibitor): Janus kinases (JAKs) are a family of enzymes involved in JAK/STAT pathway, and ruxolitinib is a pan inhibitor with more selectivity toward JAK1 and 2 (22). Ruxolitinib has been evaluated in multiple clinical trials for different types of cancer (23, 24), including breast cancer (25). It is currently under evaluation in multiple phase I and II clinical studies, including NCT01594216, NCT02876302, and NCT02928978; (b) Everolimus (EVE; selective mTORC1 inhibitor): mTOR is one of the downstream effectors of PI3K/Akt pathways, and everolimus selectively targets the C1 protein complex (26). It is approved by FDA for breast cancer treatment, and has been used in clinical settings for different types of cancer (27, 28); and (c) Erlotinib (ERL; selective ErbB1/EGFR inhibitor): Small molecules that target Erb family of receptors collectively (pan-ErbB inhibitors) and selectively, have been studied in clinical settings. Erlotinib is a selective inhibitor that targets EGFR specifically, and has been in clinical trials for treatment of advanced solid malignancies (29). Erlotinib is under extensive clinical evaluation for breast cancer treatment, including phase II clinical trials NCT00633750, NCT00054275, NCT00054132, NCT00834678, NCT00033514, and NCT00733408. EGFR has been linked to both PIEK/Akt (30) and Ras/Raf/MEK/ERK pathways (31).

It is important to note that our objective was not to investigate combination of these selected molecularly-targeted drugs in breast cancer therapy. Our goal was to identify specific “alternative” (or “off-target”) proteins with specific role in resistance against each drug independently. We selected three small molecule drugs to study resistance mechanisms against targeting proteins involved in three major signaling pathways: JAK as the triggering upstream protein in JAK/STAT signaling axis (with reported cross-talk with other pathways), mTOR as a downstream and central effector for PI3K/Akt pathway, and EGFR as a receptor tyrosine kinase (RTK) and upstream factor for both PI3K/Akt and RAS/Raf/MEK/ERK pathways.

## Materials and Methods

### Materials

Ruxolitinib, everolimus, and erlotinib were purchased from Selleckchem (Houston, TX). Dulbecco's modified Eagle's medium (DMEM; low glucose with L-glutamine), penicillin (10,000 U/mL), streptomycin (10 mg/mL), fetal bovine serum (FBS), and Hank's Balanced Salt Solution (HBSS) were provided by Life Technologies (Grand Island, NY). PCR master mixes (iScriptTM Reverse transcription Supermix and iTaq Universal SYBR Green Supermix), and all Western Blot requirements (including Trans-blot® TurboTM Cassettes, Trans-Blot® Turbo™ Mini PVDF Transfer Packs, Clarity™ Western ECL Substrate, and 10% Mini-PROTEAN® TGX Stain-Free™ Protein Gels) were were purchased from Bio-Rad (Hercules, CA). Cell Counting Kit-8 (CCK-8) assay was supplied by Biotool (Houston, TX). TRIzol reagent was purchased from Sigma (St. Louis, MO). The Pierce™ Bovine Serum Albumin Standard Ampules were obtained from ThermoFisher Scientific (Waltham, MA). Monoclonal antibodies against AKT1 (2H10 Mouse mAb; Cat# 2967, RRID:AB_331160), MAPK7 (Erk5 (D3I5V) Rabbit mAb; Cat# 12950, RRID:AB_2798068), and β-actin (8H10D10 Mouse mAb; Cat# 3700, RRID:AB_2242334), and anti-rabbit polyclonal secondary antibody HRP-linked (Cat# 7074, RRID:AB_2099233) were provided by Cell signaling Inc. (Danvers, MA). Monoclonal antibodies for JAK3 (clone 452506; Cat# MAB4699, RRID:AB_2128788), RPS6KA5 Human MSK1 MAb (clone 252608; Cat# MAB2518, RRID:AB_2181800), and Bcl-2 (Human/Mouse/Rat Bcl-2 MAb Clone 625509; Cat# MAB8272, RRID:AB_10890789), fluorescent-labeled monoclonal antibodies for HER2 (Human ErbB2/Her2 (Trastuzumab) Alexa Fluor 488 MAb Cl Hu5; Cat# FAB9589G, RRID:AB_2800468), and EGFR (Human EGF R/ErbB1 Fluorescein-conjugated Antibody; Cat# FAB10951F, RRID:AB_1096584), and anti-mouse polyclonal secondary antibody (Goat Anti-Mouse IgG HRP-linked; Cat# HAF007, RRID:AB_357234) were purchased from R&D Systems (Minneapolis, MN). All other materials were obtained from VWR (Radnor, PA). All the primers were designed and ordered from Integrated DNA Technologies (IDT; Coralville, IA). Supplementary Table 1 summarizes the sequence of the primers included in the microarrays used in this study. All primers were validated by gel electrophoresis (to ensure exclusive amplification of the target cDNA) and real-time PCR using differtent cDNA concentrationsm as described before (32). All the siRNAs used for the study were purchased from Qiagen (Valencia, CA), and the relevant information is summarized in Supplementary Table 2.

### Cell Lines

Three different breast cancer cell lines were included in this study:

- MDA-MB-231 wild-type (triple negative, but expected to express EGFR; ATCC® HTB-26™, RRID:CVCL_0062);
- MDA-MB-468 (triple negative, but over-expresses EGFR; ATCC® HTB-132™, RRID:CVCL_0419); and
- AU565 (over-expresses HER2, and expected to express EGFR; ATCC® CRL-2351™, RRID:CVCL_1074) (33).

The selected cell lines therefore represent a variety of receptor expression, and as a result, a range of active signaling pathways. More detailed information about the selected cell lines are summarized in Supplementary Table 3. All cell lines were incubated for the duration of experiments in 37°C and 5% CO_2_ level. Dulbecco's Low Glucose Modified Eagles Medium (DMEM), containing L-Glutamine and Sodium Pyruvate and glucose was used for MDA-MB-231 and MDA-MB-468 cells. RPMI 1640 medium was used for AU565 cells. Both mediums were supplemented with 10% (v/v) fetal bovine serum, 100 U/mL penicillin and 100 μg/ml streptomycin.

### Cell Viability Assay

A Cell Counting 8 (CCK8) KIT (Biotool; Houston, TX; also known as WST-8) was used to evaluate the Lethal concentration 50% (LC50) of molecularly-targeted drugs. After the treatment period, 10 μL of CCK8 reagent was added to each well, and plates were incubated at 37°C, 5% CO_2_ for 2 h. Absorbance of each well was measured at 450 nm using microplate reader (SpectraMAX M5 microplate reader). The results were normalized to cells treated with normal saline (considered as 100%) after subtracting the signal from blank wells (medium without cells in the plate with CCK-8 solution added). All experiments were performed in triplicates.

### LC50 Determination

Cells were seeded in 96-well plates (~10^5^ cells per well) and were incubated at 37°C, 5% CO_2_ for 24 h. After the incubation period, cells were exposed to a range of drug concentrations in triplicates, and were incubated in the same conditions. After 72 h exposure, CCK assay was performed. We estimated LC50 values using sigmoidal effect (% cell death) model according to the following equation:

$$\begin{array}{l} \%\;Cell\;Death=\;\frac{\%\;Maximum\;Cell\;Death\;\times \;C^{\gamma }}{{LC50}^{\gamma }\;+\;C^{\gamma }} \end{array}$$

where C is the concentration of drug, LC50 is the concentration that produces half of the maximum cell death, and γ is the hill coefficient (steepness factor). The experimental values of % cell death and concentration were fitted to the above equation using non-linear regression analysis and the values of % maximum cell death, LC50, and γ were estimated. The values for Maximum cell death, γ, and LC50 are summarized in Supplementary Table 4.

### Inherent and Acquired Resistance

Three approaches were used to either collect the inherently non-responsive cells or to induce resistance in naïve cells: (i) Single shock method: Cells were exposed to a high concentration of one of the selected molecularly-targeted drugs, equivalent to 4 × LC50 estimated for each cell line. After 24 h, the medium containing the drug was replaced with fresh, drug-free medium. After 72 h of the initial exposure, surviving cells were collected, and transferred to a cell culture flask, with medium containing the respective drug equivalent to 50% of LC50 as the maintenance concentration. The inherent resistance was confirmed by LC50 determination in surviving cells. The total RNA was extracted from a sample subset of the surviving population; (ii) “Multiple Exposures”: Cells were exposed to concentrations equivalent to 1.5 × LC50 calculated for the drug in each cell line. Exposure was repeated until a ~3-fold increase in LC50 value was observed. After each exposure surviving cells were collected and the LC50 of the drug in the surviving population was determined. The total RNA was extracted for the resistant cells, and cells were cultured in medium containing the same maintenance concentration; and (iii) “Gradual Method”: Exposures started with concentrations equivalent to 10% of the LC50 calculated for the drug in each cell line. Each cell population was exposed to each concentration for three times, before the exposure concentration increased. The drug concentration was increased step-wise to concentrations equivalent to 20, 50, 100, and 150% (1.5 ×) LC50. Resistant cells were maintained in medium containing the maintenance concentration. LC50 was periodically determined for all resistant cell populations to ensure continuance of the elevated LC50.

### Real-Time Polymerase Chain Reaction (RT-PCR)

RNA was extracted from cells using TRIzol reagent following the manufacturer's instructions. Briefly, one mL of TRIzol reagent was added for each 1 × 10^6^ cells. The cell lysates were incubated at room temperature for 5 min, after which chloroform (at 1:5 v/v chloroform:TRIzol ratio) was added to the lysates. The contents of tubes were mixed and incubated for 2–3 min at room temperature, and then the aqueous phase was collected. Isopropanol was added to precipitate and pellet RNA using centrifuge (12,000 g for 10 min in 4°C). The pellet was washed with 75% ethanol, and extracted RNA was dissolved in RNase-free water. The total extracted RNA in each sample was determined by BioSpec-Nano (Shimadzu, Columbia, MD). For cDNA synthesis 0.5–1 μg RNA was reverse transcribed using iScriptTM reverse transcription Supermix and the C1000 Touch® thermocycler (Bio-Rad, Hercules, CA), following the manufacturer's guidelines. A CFX96TM optical module (Bio-Rad, Hercules, CA) was used for RT-PCR analysis, while human β-actin and hypoxanthine phosphoribosyltransferase 1 (HPRT1) were used as the endogenous gene to normalize the mRNA level of targeted proteins. RT-qPCR was conducted using iTaq universal SYBR green Supermix kit. Cycling conditions were as follows; 95°C for 2 min, followed by 40 cycles of denaturation 95°C for 5 s and annealing temperature 55°C for 30 s, and a final extension step at 65°C for 5 s. Analysis was performed by calculating ΔΔCT (using the average ΔCT calculated for each of the endogenous genes) and relative quantity (RQ). The mRNA levels were determined as a microarray format including 46 targeted proteins. The sequence of the primers used in this study are summarized in Supplementary Table 1.

### Western Blot

Protein expression of selected targets was analyzed by Western blot. Cell protein lysates were prepared according to standard protocol using RIPA buffer. Briefly, treated and transfected cells were collected by trypsinization, and the cell suspension was centrifuged at 800 RPM for 5 min. Then the supernatant was discarded, and the cell pellet was washed three times with ice cold PBS and then 100 μL of RIPA buffer was added to 25 μL of cell pellet and pipetted up and down. The cell lysates were then incubated on ice for 1 h, during which the tubes were sonicated (for 3 min) on ice and mixed every 10 min. The tubes centrifuged at 12,000 g for 15 min at 4°C. The supernatant was transferred to pre-cooled tubes, and total protein concentration was determined using BSA assay according to standard protocol. Briefly, 200 μL of work reagent (50:1 A:B) was added to 25 μL of standard and unknown sample in triplicate into a microplate well, plated was mixed on plate shaker for 30 s then it was incubated at 37°C in 5% CO_2_ for 30 min. Then the absorbance was measured at 562 nm using microplate reader (SpectraMAX M5 microplate reader). Protein (25 μg) was loaded per well in a 10% Mini-PROTEAN® TGX Stain-Free™ Protein gel using electrophoresis buffer (0.192 M glycine, 25 mM Tris, 0.1% SDS), and the electrophoresis was run for 30 min with 200 V. After electrophoresis, the gel was transferred onto a Trans-Blot® Turbo™ Mini PVDF membrane (Catalog No. 1704156). Membranes were blocked in BSA 5% for 3 h, and then incubated overnight (at 4°C) with the primary anti-body (1:1,000 in TBS-T). Then, the membrane was washed with TBS-T six times (5 min for each time). Then the membrane was incubated with secondary HRP-linked antibody (1:1,000 in TBS-T) for 1 h. Then the washing step was repeated. Detection was done by ECL Detect Kit using ChemiDoc imager (Bio-Rad).

### Flow Cytometry

The expression level of EGFR and HER2 was evaluated by exposing the cells to FAM-labeled antibody for the receptors of interest, and the fluorescence signal was measured by flow cytometry (BD-FACSVerse; BD Biosciences; San Jose, CA), using FITC channel. Cells were trypsinized and collected, washed with HBSS, and then re-suspend in 10 ml of staining Buffer. 200 μL of the suspension was transferred to small tubes (triplicate) and 5 μl of antibody was added to each. The Tubes were incubated in 4°C for 30 min. After incubation, tubes were centrifuged and the supernatant was discarded. Cells were washed with HBSS to remove unbound antibody and fixed by addition of 400 μL of 3.7% formaldehyde to each tube. The percentage of cells with fluorescence signal and the mean fluorescence of the cell population were calculated based on the calibration of the signal gated with non-treated cells (as the negative control).

### Protein Silencing

For silencing experiments, we included a Control siRNA (CsiRNA) group, where cells received scrambled siRNA as a negative control. Cells were seeded into six-well culture plates with antibiotic-free culture medium at 50% confluency (~6 × 10^5^ cells). After 24 h of incubation in 37°C, siRNA transfection was performed using Lipofectamine® 2000 reagent. Lipofectamine® 2000 reagent was diluted with Opti-MEM medium in 1:100 ratio. Opti-MEM medium was also used to dilute 10 nM siRNA at ratio of 1:50. After 5 min, diluted siRNA was mixed with Lipofectamine® 2000 reagent in one tube and incubated for 20 min at room temperature. Then, 500 μL of the complex solution was added to each well after removing the growth medium. Cells were incubated at 37°C for 6 h, after which, the medium containing complexes was removed and replaced by antibiotic-free culture medium for the rest of incubation time (72 h total).

## Results

### Isolation of Innately Resistant Cells and Resistance Induction

**Figure 1:**
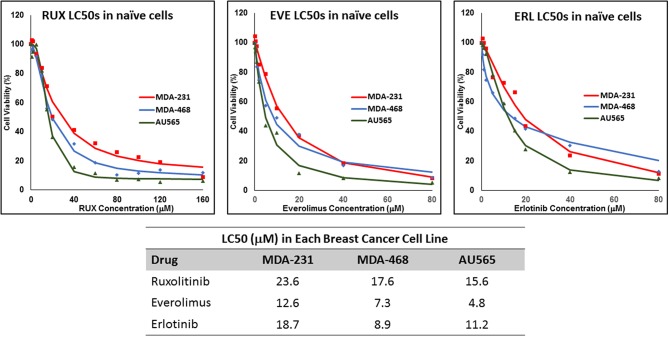
LC50 of selected molecularly-targeted drugs in naïve cells: The selected breast cancer cell lines (MDA-MB-231, MDA-MB-468, and AU565 were exposed (in triplicates) to a wide range of concentrations of ruxolitinib (RUX), everolimus (EVE), and erlotinib (ERL) and the lethal concentration for 50% cell death (LC50) was calculated based on a sigmoidal effect (% cell death) model (summarized in the table).

We determined the LC50 of selected drugs in each cell line (Figure 1) as a basis for calculation of concentrations used in future experiments. Among the selected drugs, everolimus and ruxolitinib showed the highest and lowest potency, respectively. Also, MDA-MB-231 cells showed the lowest sensitivity to the selected molecules with highest LC50 observed for all selected molecules. The lowest LC50 was calculated for everolimus in AU565 cells.

**Figure 2:**
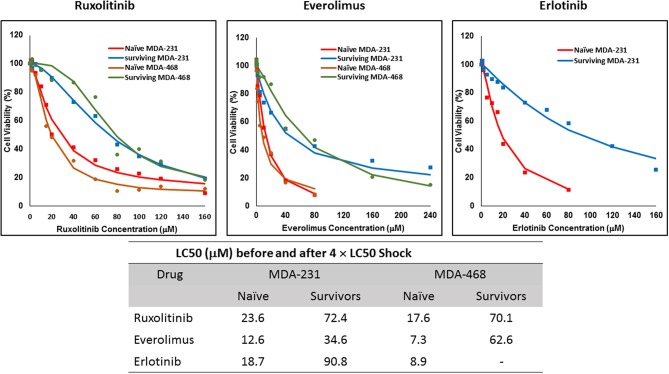
LC50 of selected molecularly-targeted drugs surviving 4 × LC50 concentrations of the respective drug: All three selected cell lines were exposed to ruxolitinib, everolimus, and erlotinib at concentrations equivalent to 4 × LC50 of the drug in each cell line. Surviving cells were collected and maintained in media containing the respective drug. The LC50 of surviving cells was calculated similarly to naïve cells, and the values are summarized in the table. We were unable to collect survivors after exposure of MDA-MB-468 cells to erlotinib and after exposure of AU565 cells to either of the selected drugs at these concentrations.

The non-responsive cells were isolated by exposing naïve cells to a concentration 4-fold the calculated LC50 in each cell line. This method was unsuccessful in AU565 cells, where we were not able to collect any survivors. We faced a similar challenge with MDA-MB-468 cells exposed to 4 × LC50 of erlotinib, where no cells were collected after exposure. The LC50 of the selected drug in the collected cells was compared to the naïve population (Figure 2), where at least a 2-fold increase in LC50 was observed. MDA-MB-231 Cells surviving the exposure to 4 × LC50 of everolimus showed the lowest difference in LC50 compared to naïve cells (~2.8-fold), where the MDA-MB-468 cells collected after same treatment with everolimus demonstrated the highest difference (~8.6-folds).

**Figure 3:**
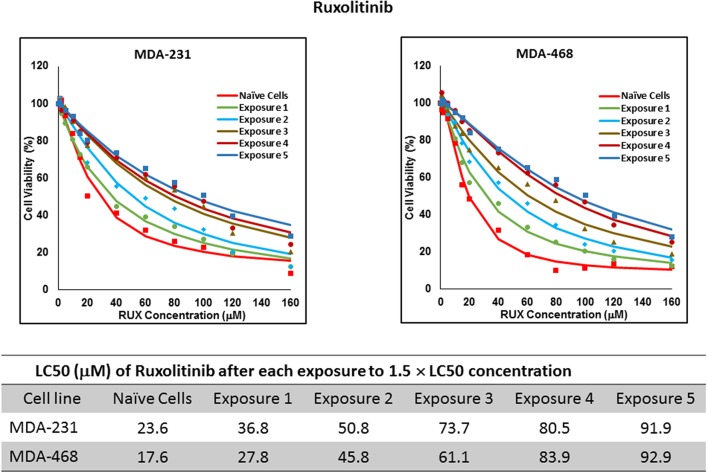
LC50 of ruxolitinib in cells surviving multiple exposure to 1.5 × LC50 concentrations of ruxolitinib. Cells were exposed to the specified concentration for total of five exposures. The LC50 of drug in corresponding surviving cell populations was calculated similarly to naïve cells, and the values are summarized in the corresponding table. We were unable to collect survivors after the first exposure of AU565 cells to ruxolitinib at these concentrations.

**Figure 4:**
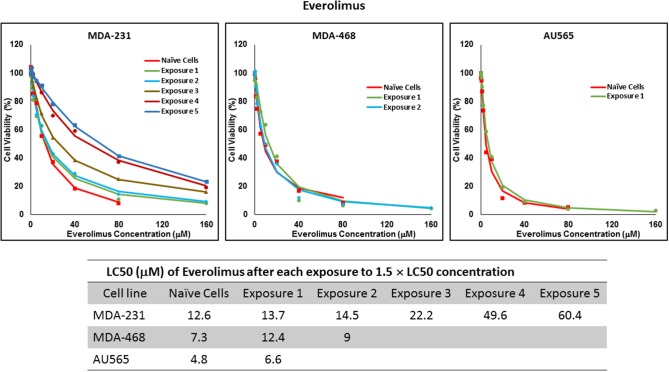
LC50 of everolimus in cells surviving multiple exposure to 1.5 × LC50 concentrations of everolimus. Cells were exposed to the specified concentration of everolimus for total of five exposures. The LC50 of drug in corresponding surviving cell populations was calculated similarly to naïve cells, and the values are summarized in the corresponding table. We were unable to collect survivors after the third exposure of MDA-MB-468 cells, and after second exposure of AU565 cells to everolimus at these concentrations.

**Figure 5:**
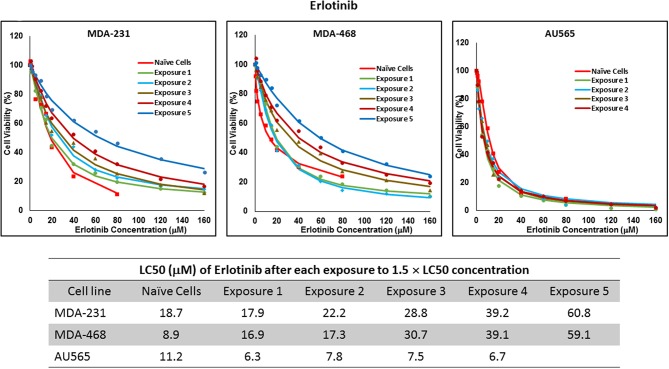
LC50 of erlotinib in cells surviving multiple exposure to 1.5 × LC50 concentrations of erlotinib. Cells were exposed to the specified concentration of erlotinib for total of five exposures. The LC50 of each drug in corresponding surviving cell populations was calculated similarly to naïve cells, and the values are summarized in the corresponding table. We were unable to collect survivors after the fifth exposure of AU565 cells to erlotinib at these concentrations.

For ruxolitinib, Multiple Exposures gradually increased the LC50 in MDA-MB-231 and MDA-MB-468 cells (to 3.9-folds and 5.3-folds after fifth exposure, respectively; Figure 3). Performing the same exposure method for everolimus was only successful in MDA-MB-231 cells (Figure 4), where a similar gradual increase was observed after 3rd exposure, and a 4.8-fold increase in LC50 was calculated after the 5th exposure. MDA-MB-468 cells only tolerated two exposures, which did not increase the LC50 significantly. For AU565 cells, we were only able to collect and grow cells after one exposure, with minimal change in LC50. Increase in LC50 was achieved for erlotinib in MDA-MB-231 and MDA-MB-468 cells using Multiple Exposures (Figure 5). Similar to our observation in everolimus experiments, exposures 1 and 2 did not affect the LC50 significantly in MDA-MB-231 cells; however, a significant increase in LC50 was observed in MDA-MB-231 and MDA-MB-468 cells after the 5th exposure (3.3- and 6.6-fold increase, respectively). AU565 cells survived four exposures without an increase in LC50.

**Figure 6:**
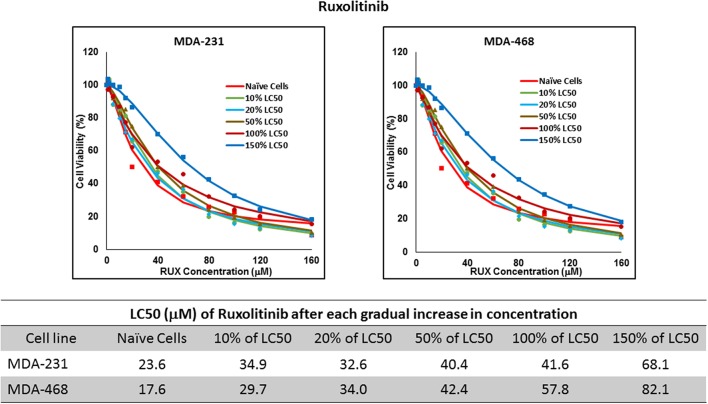
LC50 of ruxolitinib in MDA-MB-231 and MDA-MB-468 after multiple exposures to gradually increasing concentrations of the drug: All three selected cell lines were exposed to ruxolitinib at gradually increasing concentrations (starting at 10% of the estimated LC50 value and up to 1.5 × LC50; at least three times for each concentration). The LC50 in corresponding cell lines was calculated with the similar method as for naïve cells, and the values are summarized in the table. We were unable to collect AU565 cells after the third exposure to 10% of estimated LC50 value for ruxolitinib.

**Figure 7:**
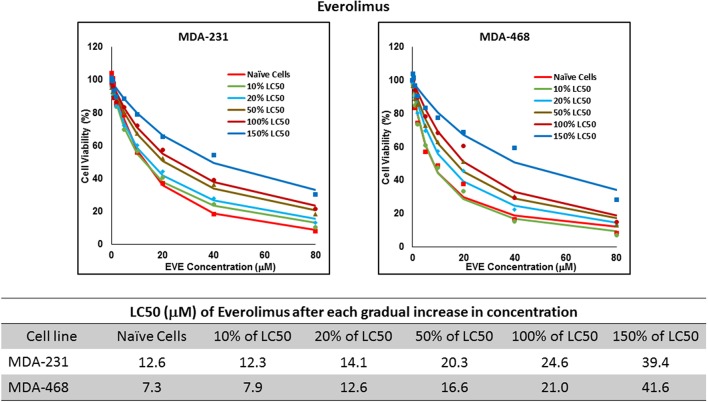
LC50 of everolimus after multiple exposures to gradually increasing concentrations of the drug: All three selected cell lines were exposed to everolimus at gradually increasing concentrations (starting at 10% of the estimated LC50 value and up to 1.5 × LC50; at least three times for each concentration). The LC50 in corresponding cell lines was calculated with the similar method as for naïve cells, and the values are summarized in the table. We were unable to collect AU565 cells after the third exposure to 10% of estimated LC50 value for everolimus.

**Figure 8:**
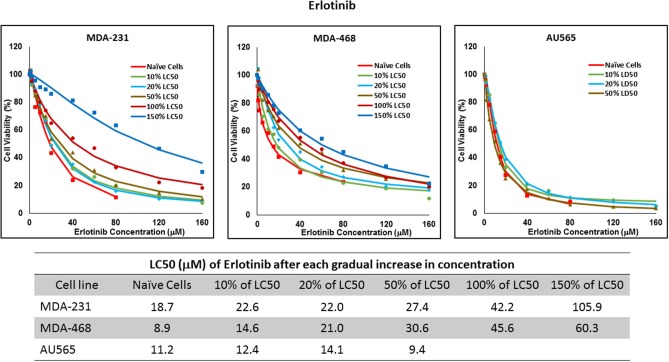
LC50 of erlotinib after multiple exposures to gradually increasing concentrations of the drug: All three selected cell lines were exposed to erlotinib at gradually increasing concentrations (starting at 10% of the estimated LC50 value and up to 1.5 × LC50; at least three times for each concentration). The LC50 in corresponding cell lines was calculated with the similar method as for naïve cells, and the values are summarized in the table. We were unable to collect AU565 cells after the third exposure to 50% of estimated LC50 value for erlotinib.

Resistance to ruxolitinib was induced in MDA-MB-231 and MDA-MB-468 cells via Gradual Method, which demonstrated a gradual increase in LC50 values after each increase in concentration, and a 2.9- and 4.7-fold increase in LC50 value was calculated after exposure to highest concentration, respectively (Figure 6). AU565 cells never truly recovered after exposures to 10% of LC50 values. A similar trend was observed in response to gradual increase in everolimus concentrations, where AU565 cells were removed from the studies, and a 3.1- and 5.7-fold increase in LC50 was observed for MDA-MB-231 and MDA-MB-468 cells, respectively (Figure 7). Gradual Method for erlotinib in MDA-MB-231 and MDA-MB-468 cells resulted in 5.7- and 6.8-fold increase in LC50 compared to naïve populations, respectively. AU565 survived gradual increase in erlotinib concentrations equivalent to up to 50% of LC50 without a significant increase in LC50 values. However, we were not able to recover the cells after repeated exposure to concentrations equivalent to LC50 value (Figure 8).

### Profiling Protein Expression

**Figure 9:**
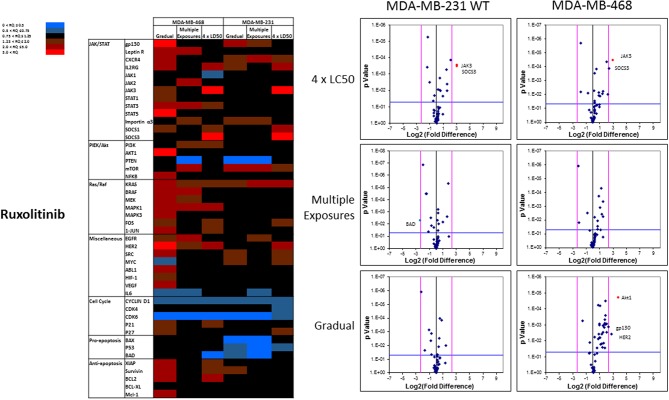
Analysis of mRNA expression level of selected proteins in resistant vs. naïve cell populations: Proteins were selected in different categories (involved in JAK/STAT, PI3K/Akt, Ras/Raf pathways, as well as pro-apoptosis, and anti-apoptosis proteins, and proteins involved in cell cycle regulation and miscellaneous proteins) and were included in a microarray along with β-actin and HPRT1 as endogenous proteins. The results for resistant cells to ruxolitinib were normalized based on relative quantities observed in corresponding naïve cell populations, and are presented as heatmaps and volcano graphs. The criteria for significant overexpression were set at 5-fold increase in mRNA level and *p* < 0.05. See Supplementary Figure 1 for corresponding scatterplots.

The mRNA levels are presented as heat maps, and scatter and volcano plots, where 5-fold increase or decrease in mRNA levels (as well as *p*-value of 0.05 for volcano plots) were selected as the cut-off to identify the most significant discrepancies. Our observations after exposure of MDA-MB-231 and MDA-MB-468 to ruxolitinib (as summarized in the heat map presented in Figure 9) indicated different adjustments in mRNA level of many selected proteins (for instance, the opposite changes in expression level of Myc in different study groups, or significant overexpression of Akt1 in MDA-MB-468 cells exposed to ruxolitinib via Gradual Method that was not seen in any other study groups). This emphasizes the different reactions to ruxolitinib exposure depending on the cell type and exposure method. However, consistencies were also detected, which could be summarized as: (i) consistent KRas overexpression across the map; (ii) consistent downregulation of cyclin D1; and CDK6 across the map; (iii) significant overexpression of JAK3 and SOCS3 in both cell lines surviving 4 × LC50 concentration; (iv) overexpression of all proteins categorized as “anti-apoptotic” (except BCL-XL) or under Ras/Raf pathway in MDA-MB-468 cells for Gradual Method; and (v) downregulation of all proteins categorized as “pro-apoptosis” in MDA-MB-231 cells repeatedly exposed to ruxolitinib (gradually increasing or equal concentrations). The volcano plots present the most significant up-and down-regulated proteins in study groups. JAK3 and SOCS3 were identified as proteins overexpressed in cells surviving 4 × LC50 of ruxolitinib in both cell lines, and while Multiple Exposure method did not identify any significant overexpression, Gradual Method resulted in significant overexpression of Akt1 mRNA in MDA-MB-468 cells.

**Figure 10:**
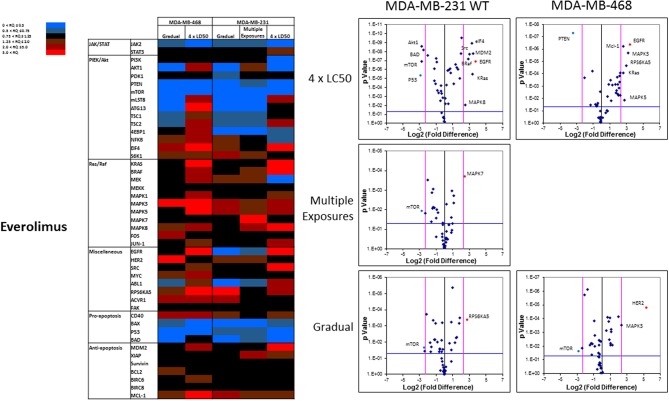
Analysis of mRNA expression level of selected proteins in resistant vs. naïve cell populations: Proteins were selected in different categories (involved in JAK/STAT, PI3K/Akt, Ras/Raf pathways, as well as pro-apoptosis, and anti-apoptosis proteins, and proteins involved in cell cycle regulation and miscellaneous proteins) and were included in a microarray along with β-actin and HPRT1 as endogenous proteins. The results for resistant cells to everolimus were normalized based on relative quantities observed in corresponding naïve cell populations, and are presented as heatmaps and volcano graphs. The criteria for significant overexpression were set at 5-fold increase in mRNA level and *p* < 0.05. See Supplementary Figure 1 for corresponding scatterplots.

The pattern of response to everolimus was different, and a higher number of discrepancies in mRNA levels was observed in study groups overall. Notable discrepancies included: (i) lower mRNA level for mTOR and PTEN, as well as pro-apoptotic proteins BAX, P53, and BAD in all study groups (except for BAD in MDA-MB-468 cells exposed to 4 × LC50 concentration of everolimus); (ii) overexpression of eIF4 (except for Multiple Exposures in MDA-MB-231 cells), S6K1 (except in cells surviving 4 × LC50 in MDA-MB-231 cells), MAPK3, MAPK5 (except in Gradual Method in MDA-MB-468 cells), RPS6KA5 (except for Multiple Exposures in MDA-MB-231 cells), and Mcl-1 in all study groups; (iii) opposite results for mRNA levels of mLST8, ATG13, TSC1, and TSC2 (overexpression or no change in cells surviving 4 × LC50 concentrations, and downregulation after Multiple Exposures); (iv) multiple significant discrepancies in survivors of 4 × LC50 in both cell lines (more than any other study group included in this study), including overexpression of EGFR in both cell lines, and downregulation of P53 and PTEN in MDA-MB-231 and MDA-MB-468 cells, respectively; (v) significant overexpression of MAPK7 and downregulation of mTOR in MDA-MB-231 cells after Multiple Exposures; and (vi) significant downregulation of mTOR in both cell lines, and overexpression of RPS6KA5 and HER2 in MDA-MB-231 and MDA-MB-468 cells, respectively (Figure 10).

**Figure 11:**
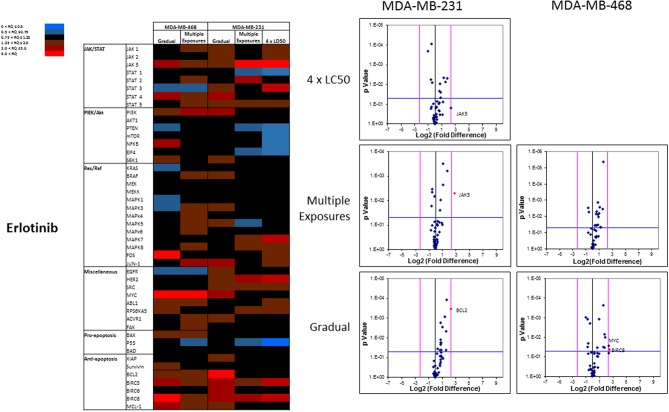
Analysis of mRNA expression level of selected proteins in resistant vs. naïve cell populations: Proteins were selected in different categories (involved in JAK/STAT, PI3K/Akt, Ras/Raf pathways, as well as pro-apoptosis, and anti-apoptosis proteins, and proteins involved in cell cycle regulation and miscellaneous proteins) and were included in a microarray along with β-actin and HPRT1 as endogenous proteins. The results for resistant cells to erlotinib were normalized based on relative quantities observed in corresponding naïve cell populations, and are presented as heatmaps and volcano graphs. The criteria for significant overexpression were set at 5-fold increase in mRNA level and *p* < 0.05. See Supplementary Figure 1 for corresponding scatterplots.

The overall number of expression discrepancies in cells exposed to erlotinib was less than our observations in cells exposed to everolimus. Significant overexpression was observed for JAK3 (Multiple Exposures in MDA-MB-231 cells), Bcl2, and BIRC8 (Gradual method in MDA-MB-231 and MDA-MB-468 cells, respectively; Figure 11). Overexpression of selected anti-apoptotic proteins in most of the study groups was another notable discrepancy.

### Sensitizing Resistant Cells by Targeting “Alterative” Protein

**Figure 12:**
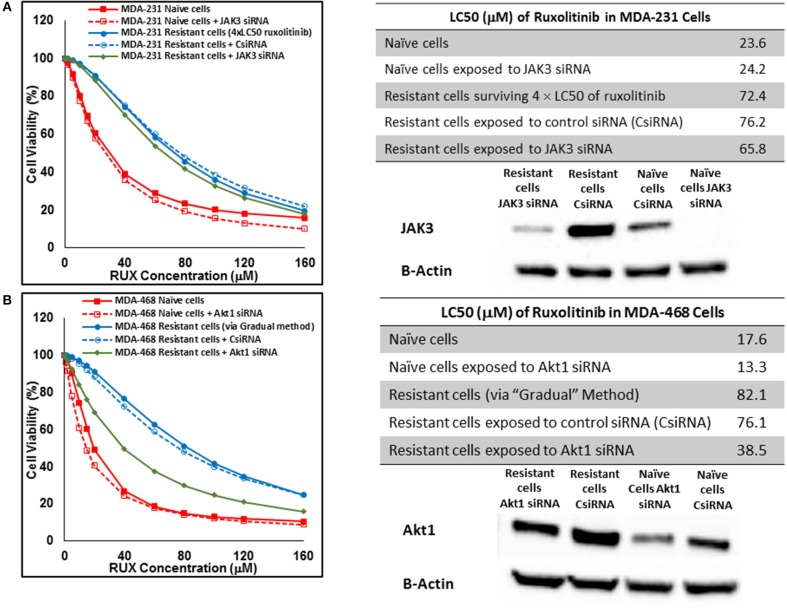
Sensitizing the resistant cells to the effect of ruxolitinib by silencing identified alternative proteins: Lipofectamine® 2000 was used for siRNA silencing of JAK3 in MDA-MB-231 cells surviving 4 × LC50 concentrations ruxolitinib **(A)**, and Akt1 in MDA-MB-468 cells with acquired resistance to ruxolitinib **(B)**. The LC50 values were calculated in naïve cells, naïve cells exposed to siRNA targeting the alternative protein, resistant cells, resistant cells exposed to scrambled siRNA, and resistant cells exposed to siRNA targeting the alternative protein. The LC50 values were calculated via the same sigmoidal effect (% cell death) model and are summarized in corresponding tables. The silencing of targeted alternative protein was confirmed via Western Blot.

Silencing of the identified proteins in resistant cells was performed via siRNA delivery to confirm the role of the non-targeted proteins in resistance against selected small molecules. JAK3 was identified as an overexpressed protein in three resistant cell populations (MDA-MB-231 and MDA-MB-468 cells surviving 4 × LC50 of ruxolitinib, and MDA-MB-231 cells surviving Multiple Exposures to erlotinib). The silencing efficiency was confirmed using Western Blot; however, it did not change the LC50 of resistant cells to the selected small molecule significantly, as compared to resistant cells exposed to scrambled (control) siRNA (CsiRNA) in any of the three cell populations (JAK3 silencing in MDA-MB-231 cells surviving 4 × LC50 of ruxolitinib is presented in Figure 12A). A similar observation was made for silencing SOCS3 in both cell lines after surviving 4 × CL50 of ruxolitinib (data not shown). Unlike JAK3 and SOCS3, silencing Akt1 (confirmed by Western Blot) in MDA-MB-468 cells exposed to gradually increasing concentrations of ruxolitinib reduced the LC50 for ruxolitinib to half (38.5 vs. 76.1 μM) for cells exposed to CsiRNA) in resistant cells (Figure 12B). Silencing Akt1 in naïve cell population had a minimal effect on ruxolitinib LC50 (13.3 vs. 17.6 μM for naïve cells).

**Figure 13:**
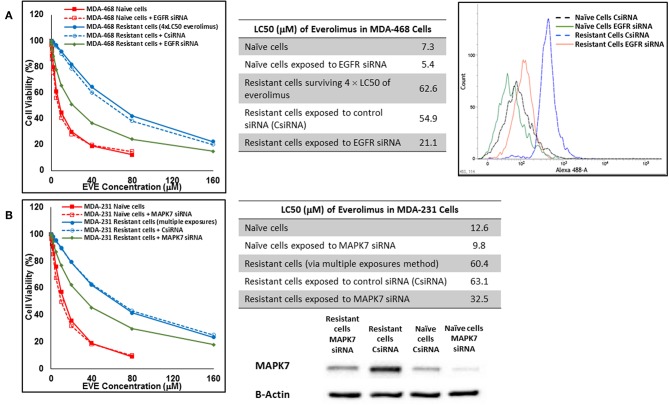
Sensitizing the resistant cells to the effect of everolimus by silencing identified alternative proteins: Lipofectamine® 2000 was used for siRNA silencing of EGFR in MDA-MB-468 cells surviving 4 × LC50 concentrations of everolimus **(A)**, and MAPK7 in MDA-MB-231 cells surviving multiple exposures to 1.5 × LC50 concentrations of everolimus **(B)**. The LC50 values were calculated in naïve cells, naïve cells exposed to siRNA targeting the alternative protein, resistant cells, resistant cells exposed to scrambled siRNA, and resistant cells exposed to siRNA targeting the alternative protein. The LC50 values were calculated via the same sigmoidal effect (% cell death) model and are summarized in corresponding tables. The silencing of targeted alternative protein was confirmed via Western Blot for MAPK7 and flowcytometry for EGFR.

**Figure 14:**
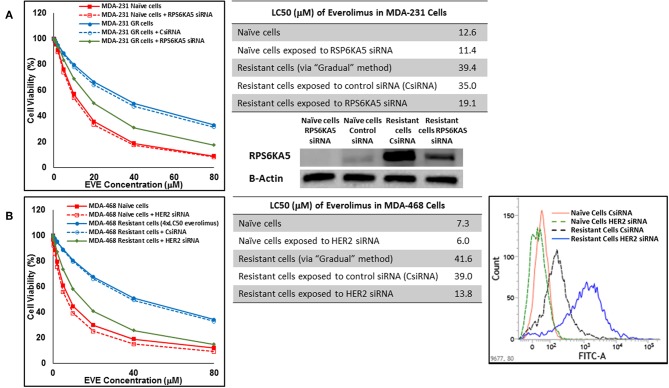
Sensitizing the cells with acquired resistance to the effect of everolimus by silencing identified alternative proteins: Lipofectamine® 2000 was used for siRNA silencing of RPS6KA5 in MDA-MB-231 cells with acquired resistance to everolimus **(A)**, and HER2 in MDA-MB-468 cells with acquired resistance to everolimus **(B)**. The LC50 values were calculated in naïve cells, naïve cells exposed to siRNA targeting the alternative protein, resistant cells, resistant cells exposed to scrambled siRNA, and resistant cells exposed to siRNA targeting the alternative protein. The LC50 values were calculated via the same sigmoidal effect (% cell death) model and are summarized in corresponding tables. The silencing of targeted alternative protein was confirmed via Western Blot for RPS6KA5 and flowcytometry for HER2.

In cells surviving 4 × LC50 of everolimus EGFR showed the most significant overexpression (in terms of fold increase) in both cell lines. EGFR silencing was confirmed by flowcytometry experiments and had minimal effect on everolimus LC50 in MDA-MB-468 naïve cells (5.4 vs. 7.3 μM). However, targeting EGFR via siRNA reduced everolimus LC50 in cells surviving 4 × LC50 by 2.6-folds (21.1 vs. 54.9 μM for resistant cells exposed to CsiRNA; Figure 13A). Silencing EGFR in MDA-MB-231 cells surviving 4 × LC50 created a similar impact on everolimus LC50 (2.2-folds reduction in LC50; data not shown). Similarly, silencing MAPK7 in naïve MDA-MB-231 cell populations had a minimal effect on LC50 (9.8 vs. 12.6 μM), and similar interference reduced everolimus LC50 by ~2-folds in resistant cells (32.5 vs. 63.1 μM in resistant cells exposed to CsiRNA; Figure 13B). Silencing RPS6KA5, while not impactful on everolimus LC50 in naïve MDA-MB-231 cells (11.4 vs. 12.6 μM), reduced everolimus LC50 by 1.8-folds (19.1 vs. 35.0 μM for resistant cells exposed to CsiRNA) in resistant cell population (Figure 14A). And finally, targeting HER2 (confirmed via flowcytometry) in MDA-MB-468 cells with induced resistance to everolimus reduced everolimus LC50 by 2.8-folds (13.8 vs. 39.0 μM for exposure to CsiRNA), with minimal effect on everolimus LC50 in naïve population (6.0 vs. 7.3 μM; Figure 14B).

**Figure 15:**
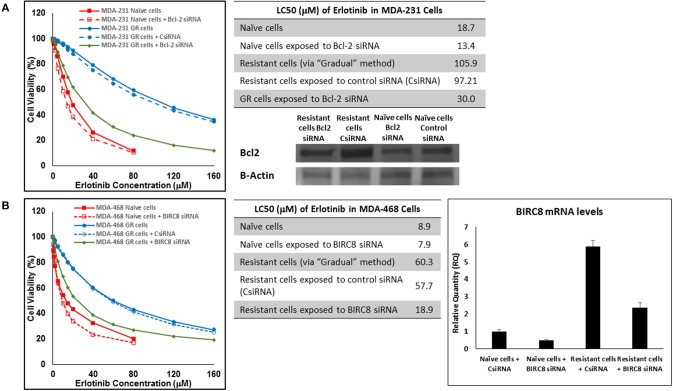
Sensitizing the resistant cells to the effect of erlotinib by silencing identified alternative proteins: Lipofectamine® 2000 was used for siRNA silencing of Bcl-2 in MDA-MB-231 cells with acquired resistance to erlotinib **(A)**, and BIRC8 in MDA-MB-468 cells with acquired resistance to erlotinib **(B)**. The LC50 values were calculated in naïve cells, naïve cells exposed to siRNA targeting the alternative protein, resistant cells, resistant cells exposed to scrambled siRNA, and resistant cells exposed to siRNA targeting the alternative protein. The LC50 values were calculated via the same sigmoidal effect (% cell death) model and are summarized in corresponding tables. The silencing of targeted alternative protein was confirmed via Western Blot for Bcl-2 and qPCR for BIRC8.

In addition to silencing JAK3 in MDA-MB-231 cells surviving Multiple Exposures to erlotinib, Bcl2 and BIRC8 were also selected for siRNA silencing in cells with induced resistance to erlotinib. Bcl2 silencing in naïve MDA-MB-231 cells did reduce erlotinib LC50 to ~72% of the original observed value (13.4 vs. 18.7 μM). It also reduced everolimus LC50 in MDA-MB-231 cells with induced resistance to erlotinib by more than 3.2-folds (30.0 vs. 97.21 μM for resistant cells exposed to CsiRNA; Figure 15A). BIRC silencing was confirmed via qPCR, since monoclonal antibody for Western Blotting was not available by either of selected vendors. Silencing BIRC8 showed more than 3-folds decrease in erlotinib LC50 in resistant cells (18.9 vs. 57.7 μL for exposure to CsiRNA), with minimal effect in naïve cells (7.9 vs. 8.9 μM; Figure 15B).

## Discussion

Three targets were selected to study the role of alternative proteins in resistance against molecularly targeted drugs targeting one or more of the major signaling pathways. JAK2 has a crucial role in cancer cell proliferation and survival, not only via activation of STATs and downstream proteins, but also through inter-pathway cross-talk to activate PI3K/AKT and MEK/ERK signaling (20, 21). EGFR is an important RTK, which is also known to trigger multiple signaling cascades (AKT, RAS/RAF, and STATs), even independent from the growth factor ligands (34). And finally, mTOR is a downstream effector of the PI3K/AKT pathway with a central role in multiple intracellular mechanisms (including autophagy, microtubule organization, and protein and lipid synthesis) (35). According to a recent study of breast cancer cell lines, while JAK/STAT pathway is not over-activated in selected cell lines for this study (compared to non-cancerous breast cancer cell lines), the activation level in AU565 cells is higher than MDA-MB-231 and MDA-MB-468 cell lines. On the other hand, PI3K/Akt pathway is over-activated in all three cell lines, with AU565 showing the highest level of overactivation (36).

mTOR is targeted by everolimus (along other small molecules; approved by FDA in breast cancer treatment) and is also one of the few intracellular proteins targeted by FDA-approved small molecule drugs (along with CDK4/CDK6 and PARP enzyme). Although ruxolitinib and erlotinib are not currently approved by FDA for breast cancer treatment, they are under investigation in multiple clinical trials for this purpose, and were included in the study since no drug targeting JAK2 or EGFR specifically (lapatinib inhibits both EGFR and HER2) is included in the FDA-approved list for breast cancer treatment at this time.

A wide range of values are usually reported for the potency of molecularly-targeted drugs, which includes cell-free assays, toxicity assays, and function assays. For instance, while 320 nM ruxolitinib was reported to eliminate the enhancing effect of G-CSF in HT93A leukemia cells (37), a ruxolitinib concentration of at least 50 μM was required to induce a significant drop in cell proliferation in hepatocellular carcinoma cell lines HuH7, SNU182, and SNU423 (38). In this study, the LC50 values in naïve cells were in micromolar range, with the least sensitivity to all three selected drugs observed in MDA-MB-231 cells. This was especially significant in LC50 values observed for erlotinib, where the LC50 value in MDA-MB-231 cells was more than 2-folds higher than the value calculated for MDA-MB-468 cells (Figure 1). This could be explained by the significantly higher expression level of EGFR in MDA-MB-468 cells compared to MDA-MB-231 cells (33).

The three selected approaches to creating resistant cell populations represent different scenarios: Using an “extremely high” concentration of the drugs (4-folds the estimated LC50 value) almost exclusively collects the cells that do not respond to the selected drugs in clinically relevant concentrations. This is confirmed by the significantly higher LC50 values in the cells that survived this concentration (Figure 2). Repeated exposures to 1.5 × LC50 concentrations, on the other hand, will most likely isolate a combination of cells with inherent and induced resistance. While a significant proportion of the sensitive cells are eliminated after each exposure, the concentration is not high enough to eliminate all the sensitive cells to the selected drug. Also, the repeated treatments provided the opportunity for the sensitive cells that were exposed to sub-effective concentrations to adapt and activate the alternative signaling. The gradual increase in estimated LC50 values indicate the gradual selection/adaptation nature of the process (Figures 3–5). A similar gradual increase in LC50 values was observed for the Gradual Method (Figures 6–8). However, this approach provided a better opportunity for the cells to adapt, by starting with significantly lower concentrations, since exposure to each concentration was repeated at least three times. The cells collected at the end of this process most probably represent cells with acquired resistance.

We were not able to collect AU565 resistant cells to none of the selected drugs. The 4 × LC50 concentration simply wiped off all the cells and repeated exposures either resulted in no surviving cells (e.g., after second exposure to everolimus; Figure 4), or did not significantly changed the LC50 in the surviving cells (e.g., after four repeated exposures to erlotinib; Figure 5). The triple-negative breast cancer cells (TNBCs) are notoriously less responsive to anticancer treatment. However, AU565 cells were included in this study for comparison to the two TNBC cell lines. Our results indicate a lower heterogeneity in the population of AU565 cells and/or a lack of plasticity to adapt to multiple exposures to selected drugs. Also, the fact that both major targeted signaling pathways are more active in AU565 cells compared to the selected TNBC cells (36), might play an important role in a higher sensitivity in this cell line to the selected small molecule drugs. And finally, different approaches to collecting inherently resistant cells and/or inducing resistance could potentially be successful in creating a resistant population of this cell line.

We analyzed the mRNA level of selected proteins. Selections were made considering the molecularly-targeted drug to which the cells were resistant, and were based on literature and our own previously reported findings. For example, SOC1 and SOC3 were included in microarrays evaluating cells resistant to ruxolitinib, due to their inhibitory effect on JAK/STAT pathway (39, 40). On the other hand, we have previously identified RPS6KA5 and ACVR1 as kinases that showed promising synergistic effect in combinatorial silencing with Mcl-1 (32).

KRas was consistently overexpressed (less than the arbitrary threshold of 5-folds increase) in cells surviving ruxolitinib exposure, and could be further studies as a potential alternative signaling avenue to compensate for JAK signaling. An interesting finding was the overexpression of SOCS1 and SOCS3 in cells with inherent resistance to ruxolitinib. This indicates that lack of response to ruxolitinib could be due to non-active JAK/STAT signaling (as opposed to overwhelming overactive pathway). In other words, cells do not respond to the molecularly-targeted drug, since the target is simply not important in the survival of this specific population of cells. A similar pattern was observed for JAK3 (Figure 9). Springuel et al. have reported a “cooperation” between JAK1 and JAK3 mutants, which results in resistance to JAK1/2 inhibitors in TS1 cells (41). Our results indicate overactivation of JAK3 in cells inherently resistant to ruxolitinib, and in fact, SOCS3 and JAK3 were the only proteins in the microarray that were overexpressed beyond the 5-fold increase threshold in this population. Interestingly, targeting neither of these proteins via siRNA silencing sensitized the cells to ruxolitinib (Figure 12A). This is certainly expected in the case of SOCS3, since silencing this inhibitory mechanism of JAK/STAT axis would only further activate the targeted signaling. JAK3 is known as a potential target for immunosuppression and cooperation with JAK1 (42), and our results indicate that JAK3 overexpression does not play a direct role in resistance to ruxolitinib. Perhaps more interestingly, JAK3 overexpression was also observed in cells surviving 4 × LC50 concentration and Multiple Exposures to Erlotinib. However, silencing JAK3 these cells did not sensitize them to the effect of Erlotinib either.

Akt1 was selected as the target with highest level of overexpression in MDA-MB-468 cells with acquired resistance to ruxolitinib. Unlike our observation with JAK3 and SOCS3, silencing Akt1 significantly reduced the LC50 in the resistant cells. Multiple reports indicate the role of JAK2 in activation of Akt signaling (43, 44). A cross-talk between STAT5 (activated by JAK2) and Akt1 has also been reported that is essential for progression of breast cancer (45). Our results indicate a compensatory role for Akt1 for cell survival after JAK inhibition via ruxolitinib.

EGFR was overexpressed in both cell lines in cell population surviving 4 × LC50 of everolimus. EGFR is known to activate both PI3K/Akt and Ras/Raf/MEK/ERK pathways, and this overexpression in cells non-responsive to everolimus might indicate an overactivation of Ras/Raf/MEK/ERK axis as an alternative signaling to mTOR. This speculation is further confirmed by the significant overexpression of BRaf, KRas, and MAPK8 in MDA-MB-231 cells and KRas, MAPK3, and MAPK5 in MDA-MB-468 cells with inherent resistance to everolimus. Downregulation of mTOR and Akt1 in resistant MDA-MB-231 cells is also another indication of relatively inactivated PI3K/Akt pathway in favor of Ras/Raf/MEK/ERK pathway in this sub-population. Silencing EGFR sensitized these cells to everolimus. After surviving Multiple Exposures to everolimus, MDA-MB-231 cells overexpressed MAPK7, which is again an indication of activation of Ras/Raf/ERK/MEK axis. Silencing MAPK7 reduced LC50 to approximately half in resistant cells. Also, after induction of acquired resistance to everolimus, mTOR was downregulated in both cell lines, which indicates inactivation of this signaling pathway in favor of alternative mechanisms. These cell lines showed different overexpression profiles. RPS6KA5 was overexpressed significantly in MDA-MB-231 cells. We have previously reported a synergistic effect for this kinase and anti-apoptotic Mcl-1 (32, 46, 47). HER2 was identified as the most significant overexpression in MDA-MB-468 cells with acquired resistance to everolimus. Similar to EGFR, HER2 is known to activate both PI3K/Akt and Ras/Raf/MEK/ERK pathways, and overexpression of HER2 seems to work as a compensating signaling pathway in acquired resistance to everolimus in a cell line that is not known for HER2 expression. Silencing RPS6KA5 and HER2 were also effective in sensitizing resistant cells to everolimus.

Anti-apoptotic proteins played a more important role in acquired resistance against erlotinib (Bcl2 and BIRC8 in MDA-MB-231 and MDA-MB-468, respectively). The role of anti-apoptotic proteins in resistance against molecularly-targeted drugs has been reported. In 2006, Schulze-Bergkamen et al. reported sensitizing hepatocellular carcinoma cells by siRNA silencing of Mcl-1 to several cytotoxic and molecularly-targeted agents, including AG1478 as an EGFR inhibitor (48). Our experiments showed a significant reduction in erlotinib LC50 in the selected cell lines after targeting Bcl2 and BIRC8 as well, which is aligned with other researchers' observations.

Overall, our data reveals involvement of a variety of alternative proteins involved in innate and acquired resistance to molecularly-targeted drugs. It has previously reported that in cancer cells sensitive to RTK inhibitors, PI3K signaling is initially lost; however, cells become resistant by finding other routes to activate PI3K signaling (49). These alternative signaling pathways are either innately overactivated in lieu of targeted protein (indicating heterogeneity responsible for innate non-responsiveness of a subpopulation of the cells), or are overactivated as an intracellular modification as a reaction to the molecularly-targeted drug (representing the plasticity of the cancer cells involved in acquired resistance). Our data also indicates that targeting well-selected alternative proteins could potentially sensitize the resistant cells to the effect of the original molecularly-targeted drug.

## Data Availability Statement

All datasets generated for this study are included in the manuscript/Supplementary Files.

## Author Contributions

All authors listed have made a substantial, direct and intellectual contribution to the work, and approved it for publication.

### Conflict of Interest

The authors declare that the research was conducted in the absence of any commercial or financial relationships that could be construed as a potential conflict of interest.
