# Supplementary material for: Heterogeneity and Plasticity of Human Breast Cancer Cells in Response to Molecularly-Targeted Drugs
Source: Front Oncol. 2019 Oct 15;9:1070. doi: 10.3389/fonc.2019.01070 (PMC6803545; doi:10.3389/fonc.2019.01070)
Supplement: Supplementary file 1 [file Data_Sheet_1.pdf]

Supplementary Table 1. The primer sets included in the microarrays used in this study

Target	Forward Sequence	Reverse Sequence
ABL1	CTC CGG GTC TTA GGC TAT AA	GTT GAC TGG CGT GAT GTA G
ACVR1	TGAGCAATGGTATAGTGGAGGATTAC	CAGACTACCTTCCTCATATCTTCAAAAC
AKT1	CAT CAG ATG AGG AGA ATG GG	GGA AGT CGC TGG TGT TAA G
ATG13	CAA CCT GCA CCC CTT CTG	TTT CGG ACA AAG ATG GTC TCC
BAD	AAC GCA GAT GCG GCA AA	TGG AGT TTC GGG ATG TGG A
BAX	CAC TGA AGC GAC TGA TGT C	TCA GCC CAT CTT CTT CCA
β-Actin	CAC ACA GGG GAG GTG ATA GC	GAC CAA AAG CCT TCA TAC ATC TCA
BCL2	GCG GCC TCT GTT TGA TTT	TCA CTT GTG GCC CAG ATA
BIRC3	GCT CGT GCT GGT TTC TAT	CAG TAG GAC TGT CTC CTC TT
BIRC6	GTG CTG ATT ATG GGA CCT AC	GTC TGT GAT CCG TTC TTC TC
BIRC8	GCT GAA GCA GTT GAC AGA	CAC ACC CAC TGT AGA GTT AC
BRAF	CAG CGA GAA AGG AAG TCA TC	TCT GCC CAT CAG GAA TCT
CD40	AAC TGT AGA TTG TGT GCA GGC TA	AGG CAA TGT TCA GCT TCA GA
CDK4	GAT CTG ATG CGC CAG TTT CTA	CCA CCA CTT GTC ACC AGA AT
CDK6	GAC CAG CAG CGG ACA AAT A	TGA CGA CCA CTG AGG TTA GA
CXCR4	GCC TTA TCC TGC CTG GTA TT	ATG AGG ATG ACT GTG GTC TTG
Cyclin D1	GCG GAG GAG AAC AAA CAG AT	GAG GGC GGA TTG GAA ATG A
4EBP1	AAT AGC CCA GAA GAT AAG CGG	ACT TTC ATA AGG CCT GGC TG
EGFR	GCG CTA CCT TGT CAT TCA	CGT CGT CCA TGT CTT CTT C
EIF4	GAG GAC GAT GGC TAA TTA CAT TGA	GCA CAG AAG TGT CTC TAG CCA AAA
FAK	CTC TCT CGA GGC AGT ATT GA	GCT GCA GGA TCT GGT TTA C
FOS	CTG AAG ACC GAG CCC TTT GA	GGA GCG GGC TGT CTC AGA
gp-130	GGG AGT GCT GTT CTG CTT TA	GTG ACC ACT GGG CAA TAT GA
HER2	ATA TGT CTC CCG CCT TCT	TTC CCG GAC ATG GTC TAA
HIF-1	CCA ACC TCA GTG TGG GTA TAA G	TTT GAT GGG TGA GGA ATG GG
HPRT1	TCT GTG GCC ATC TGC TTA	AGG AAT GCA GCA ACT GAC
IL2RG	ACC ACT GTT TGG AGC ACT T	GTT TCT GCC CAT CCA CAC TA
IL6	AAA GAG GCA CTG GCA GAA A	CAG GCA AGT CTC CTC ATT GAA
Importin α3	TCA GCC ACC AGG AAG TTA AAG	TGC TGG GAA GTG TGA AAG AG
JAK1	CTA CCG GAT GAG GTT CTA TTT C	GGG TTG CAT CTG GAA TCT T
JAK2	AAC TGC AGA TGC ACA TCA TTA CCT	TCG AAA TTG GGC CAT GAC A
JAK3	ACC AGC AGA GGG ACT TT	CAT AGC TGA CAC CAC GAT AC
JUN1	GAG AGG AAG CGC ATG AGG AA	TCC AGC CGG GCG ATT
KRAS	GCT CAG GAC TTA GCA AGA AG	GCA TCA TCA ACA CCC TGT
LEPTIN R	TCA GCT ACA TCC CTG CTA GT	TCC AGT CAC TCC AGA TTC CT
MAPK1	GTA CAG GAC CTC ATG GAA AC	CCT CTG AGG ATC TGG TAG AG
MAPK3	CTG AAC TCC AAG GGC TAT AC	TGA GCT GAT CCA GGT AGT
MAPK4	ACC TGG ACG TGT TCA TCT	TGA GGT CGC CCA GTT TAT
MAPK5	CCG GCA CTT TAC AGA GAA G	TTC AGG CTT GAG GTC TCT
MAPK6	GGA GAC AGA CTT GGC TAA TG	GCT GGT TTG AGA TCT CTG TG
MAPK7	CGC ATT AAG GAG GCC ATT	GGA CTG GGC ATT TCA ACA
MAPK8	GGT GCA TCA TGG GAG AAA	TGG TGT TCC AAG CTG TTC
MEK	AGC TGG AGC TGA TGT TTG	CCA TTC CGT ATG AGC TAA GG
MEKK	GAG ACA GCC CAG ACA ATA AA	CCC GGA GCA TCA CAA ATA G
MCL-1	CCT TTG TGG CTA AAC ACT TGA AG	CGA GAA CGT CTG TGA TAC TTT CTG
MDM2	GCA GTG AAT CTA CAG GGA CG	GTG CAT TTC CAA TAG TCA GCT AAG
mLST8	AT CCA CAT CTG GGA CTT GA	ATT CCA GAC ATA GCA GTT TCC
mTOR	GGC CGG AGT GTT AGA ATA TG	CTT GTC ATA GGC CAC AAG G

MYC	TGG TCT TCC CCT ACC CTC TCA	AGA ATC CGA GGA CGG AGA GAA
NFĸB	AAT GGG CTA CAC CGA AGC AA	CAG CGA GTG GGC CTG AGA
PDK1	ACA TGG TTG CAG GTC TCT AGT TT	AAG TAC TGA ACA TTC TGG CTG GT
PI3K	CCG AAA GGG TGC TAA AGA G	TGA GGT ACT GGC CAA AGA
PTEN	GGG ACG AAC TGG TGT AAT G	GAA ATC TAG GGC CTC TTG TG
P21	GCG ACT GTG ATG CGC TAA T	GTG GTG TCT CGG TGA CAA AG
P27	GAG CAA TGC GCA GGA ATA AG	TCC ACA GAA CCG GCA TTT
P53	GCG TGT GGA GTA TTT GGA TG	TGT AGT GGA TGG TGG TAC AG
RPS6KA5	GAC ACT GCA GCC CAG CAA	CCT AAG CTA CTG AGT CCG AGA ACTG
SOCS1	CGC CCT TAG CGT GAA GAT G	GCT GCC ATC CAG GTG AAA
SOCS3	CCA CTC TTC AGC ATC TCT GTC	TCG TAC TGG TCC AGG AAC T
STAT1	GTT CAC TAT AGT TGC GGA GAG	AGG GTC ATG TTC GTA GGT
STAT2	AGG AAA GGG CAG CAA TAA G	CTT CAG ACC CTG GTA GGT AT
STAT3	ACA ACA TGT CAT TTG CTG AAA TCA	TCC TTG GGA ATG TCA GGA TAG AG
STAT4	GAA AGC CAT CTC GGA GGA ATA A	CGG CCT TTA TTG TAG GGT TCT
STAT5	TTC TGG CAG TGG TTT GAC	GTG GGC CTG TTG CTT ATT
SRC	CAG GCT GAG GAG TGG TAT T	CCT TTC GTG GTC TCA CTT TC
Survivin	CCC CTC GGG CCA ACT G	CAG TTT GGC TTG CTG GTC TCT
S6K1	AAT CCG ATC ACC TCG AAG ATT TAT	CTG TGC TGG CCG AAG CA
TSC1	GCA AAA GGA AAC ACA GAG GAA G	AGA CTT GCT GGG TAA AGG C
TSC2	TCG CAA GGA TGG TTC AGA TG	GAC AGA GGG TAA CGA TGA ACA G
VEGF	TGC TGT CTT GGG TGC ATT	ATT CTG CCC TCC TCC TTC T
XIAP	CGT GCG GTG CTT TAG TT	GAC TGC GTG GCA CTA TTT
