# Supplementary material for: Heterogeneity and Plasticity of Human Breast Cancer Cells in Response to Molecularly-Targeted Drugs
Source: Front Oncol. 2019 Oct 15;9:1070. doi: 10.3389/fonc.2019.01070 (PMC6803545; doi:10.3389/fonc.2019.01070)
Supplement: Supplementary file 2 [file Data_Sheet_2.pdf]

Supplementary Table 2. Information regarding the siRNAs used for the protein silencing experiments

Target	Catalogue Number	Sequence
AKT1	SI00299145	Sense: 5'-UCACACCACCUGACCAAGATT-3'
		Antisense: 5'-UCUUGGUCAGGUGGUGUGATT-3'
BCL2	SI00299397	Sense: 5'-CAUUAUAAGCUGUCGCAGATT-3'
		Antisense: 5'-UCUGCGACAGCUUAUAAUGGA-3'
BIRC8	SI00146202	Sense: 5'-CGAGGUGCUCACUGCGCAATT-3'
		Antisense: 5'-UUGCGCAGUGAGCACCUCGTG-3'
EGFR	SI00300104	Sense: 5'-GCUCACGCAGUUGGGCACUTT-3'
		Antisense: 5'-AGUGCCCAACUGCGUGAGCTT-C'
EIF4	SI00300125	Sense: 5'-GAGCGGCUCCACCACUAAATT-3'
		Antisense: 5'-UUUAGUGGUGGAGCCGCUCTT-3'
HER2	SI00300195	Sense: 5'-GUGUGCACCGGCACAGACATT-3'
		Antisense: 5'-UGUCUGUGCCGGUGCACACTT-3'
JAK3	SI00604800	Sense: 5'-GGAGAUUCAGAUCCUCAAATT-3'
		Antisense: 5'-UUUGAGGAUCUGAAUCUCCCG-3'
MAPK7	SI00606039	Sense: 5'-GACCCACCUUUCAGCCUUATT-3'
		Antisense: 5'-UAAGGCUGAAAGGUGGGUCTG-3'
RPS6KA5	SI02225433	Sense: 5'-GCCAGUCAUUCGAGAUGAATT-3'
		Antisense: 5'-UUCAUCUCGAAUGACUGGCTT-3'
SOCS3	SI00058345	Sense: 5'-GGGAGUUCCUGGACCAGUATT-3'
		Antisense:5'-UACUGGUCCAGGAACUCCCGA-3'
