# Supplementary material for: Heterogeneity and Plasticity of Human Breast Cancer Cells in Response to Molecularly-Targeted Drugs
Source: Front Oncol. 2019 Oct 15;9:1070. doi: 10.3389/fonc.2019.01070 (PMC6803545; doi:10.3389/fonc.2019.01070)
Supplement: Supplementary file 3 [file Data_Sheet_3.docx]

**Supplementary Table 3.** A detailed comparison between the selected breast cancer cell line in this study

| **Cell Line** | ER | PR | HER2 | EGFR | Type | Morphology | Tumorigenic | Metastasis | Reference |
| --- | --- | --- | --- | --- | --- | --- | --- | --- | --- |
| **MDA-MB-231** | No | No | 0-1+ | 1+ | Basal | Epithelial | Yes | Yes | 1,2,3 |
| **MDA-MB-468** | No | No | 0 | 3+ | Basal | Epithelial | Yes | Yes | 1,2,3 |
| **AU565** | 0 | 0 | 3+ | 1+ | Luminal | Epithelial | Yes | Yes | 1,2,3 |

EGFR: Epithelial Growth Factor Receptor; ER: Estrogen Receptor; HER2: Human Epidermal growth factor Receptor 2; PR: Progesterone Receptor

* The extent of expression for ER, PR, HER2, and EGFR is expressed as Allred scores, as reported in reference 1 below.

1. Subik K, Lee JF, Baxter L, Strzepek T, Costello D, Crowley P, et al. The Expression Patterns of ER, PR, HER2, CK5/6, EGFR, Ki-67 and AR by Immunohistochemical Analysis in Breast Cancer Cell Lines. Breast Cancer: Basic and Clinical Research. 2010;4:35-41.

2. Smith SE, Mellor P, Ward AK, Kendall S, McDonald M, Vizeacoumar FS, et al. Molecular characterization of breast cancer cell lines through multiple omic approaches. Breast Cancer Research. 2017;19:65.

3. American Type Culture Collection (ATCC). <https://www.atcc.org/> Accessed 9/15/2019.
