# Supplementary material for: Heterogeneity and Plasticity of Human Breast Cancer Cells in Response to Molecularly-Targeted Drugs
Source: Front Oncol. 2019 Oct 15;9:1070. doi: 10.3389/fonc.2019.01070 (PMC6803545; doi:10.3389/fonc.2019.01070)
Supplement: Supplementary file 4 [file Data_Sheet_4.docx]

**Supplementary Table 4.** Parameters of the sigmoidal model for LC50 calculations

| **Small Molecule** | **Cell Line - Treatment** | **Max. Effect (% Death)** | **γ** | **LC50 (μM)** |
| --- | --- | --- | --- | --- |
| Ruxolitinib | MDA-231 - NT | 89.33 | 1.47 | 23.6 |
|  | MDA-231 - 4 × LD50 | 100 | 1.79 | 72.4 |
|  | MDA-231 - 150% LD50 (1^st^ Exposure) | 100 | 1.09 | 36.8 |
|  | MDA-231 - 150% LD50 (2^nd^ Exposure) | 100 | 1.25 | 50.8 |
|  | MDA-231 - 150% LD50 (3^rd^ Exposure) | 100 | 1.23 | 73.7 |
|  | MDA-231 - 150% LD50 (4^th^ Exposure) | 100 | 1.18 | 80.5 |
|  | MDA-231 - 150% LD50 (5^th^ Exposure) | 100 | 1.14 | 91.9 |
|  | MDA-231 - 10% (Gradual) | 100 | 1.46 | 34.9 |
|  | MDA-231 - 20% (Gradual) | 100 | 1.31 | 32.6 |
|  | MDA-231 - 50% (Gradual) | 100 | 1.50 | 40.4 |
|  | MDA-231 - 100% (Gradual) | 100 | 1.17 | 41.6 |
|  | MDA-231 - 150% (Gradual) | 100 | 1.70 | 68.1 |
|  | MDA-468 - NT | 91.58 | 1.69 | 17.6 |
|  | MDA-468 - 4 × LD50 | 85.44 | 3.15 | 70.1 |
|  | MDA-468 - 150% LD50 (1^st^ Exposure) | 94.06 | 1.33 | 27.8 |
|  | MDA-468 - 150% LD50 (2^nd^ Exposure) | 100 | 1.26 | 45.8 |
|  | MDA-468 - 150% LD50 (3^rd^ Exposure) | 100 | 1.25 | 61.1 |
|  | MDA-468 - 150% LD50 (4^th^ Exposure) | 100 | 1.43 | 83.9 |
|  | MDA-468 - 150% LD50 (5^th^ Exposure) | 100 | 1.36 | 92.9 |
|  | MDA-468 - 10% (Gradual) | 98.07 | 1.27 | 29.7 |
|  | MDA-468 - 20% (Gradual) | 99.62 | 1.19 | 34.0 |
|  | MDA-468 - 50% (Gradual) | 100 | 1.19 | 42.4 |
|  | MDA-468 - 100% (Gradual) | 100 | 1.30 | 57.8 |
|  | MDA-468 - 150% (Gradual) | 100 | 1.66 | 82.1 |
|  | AU565 - NT | 92.63 | 3.01 | 15.6 |
| Everolimus | MDA-231 - NT | 100 | 1.26 | 12.6 |
|  | MDA-231 - 4 × LD50 | 88.86 | 1 | 34.6 |
|  | MDA-231 - 150% LD50 (1^st^ Exposure) | 100 | 1 | 13.7 |
|  | MDA-231 - 150% LD50 (2^nd^ Exposure) | 98.98 | 1 | 14.5 |
|  | MDA-231 - 150% LD50 (3^rd^ Exposure) | 95.7 | 1 | 22.2 |
|  | MDA-231 - 150% LD50 (4^th^ Exposure) | 100 | 1.17 | 49.6 |
|  | MDA-231 - 150% LD50 (5^th^ Exposure) | 100 | 1.22 | 60.4 |
|  | MDA-231 - 10% (Gradual) | 100 | 1 | 12.3 |
|  | MDA-231 - 20% (Gradual) | 99.21 | 1 | 14.1 |
|  | MDA-231 - 50% (Gradual) | 99.51 | 1 | 20.3 |
|  | MDA-231 - 100% (Gradual) | 100 | 1 | 24.6 |
|  | MDA-231 - 150% (Gradual) | 100 | 1 | 39.4 |
|  | MDA468 - NT | 95.82 | 1 | 7.3 |
|  | MDA-468 - 4 × LD50 | 100 | 1.32 | 62.6 |
|  | MDA-468 - 150% LD50 (1^st^ Exposure) | 100 | 1.21 | 12.4 |
|  | MDA-468 - 150% LD50 (2^nd^ Exposure) | 100 | 1.04 | 9.0 |
|  | MDA-468 - 10% (Gradual) | 99.44 | 1 | 7.9 |
|  | MDA-468 - 20% (Gradual) | 98.93 | 1 | 12.6 |
|  | MDA-468 - 50% (Gradual) | 100 | 1 | 16.6 |
|  | MDA-468 - 100% (Gradual) | 100 | 1.10 | 21.0 |
|  | MDA-468 - 150% (Gradual) | 100 | 1 | 41.6 |
|  | AU565 - NT | 99.60 | 1.15 | 4.8 |
|  | AU565 - 150% LD50 (1st Exposure) | 100 | 1.18 | 6.6 |
| Erlotinib | MDA-231 - NT | 100 | 1.37 | 18.7 |
|  | MDA-231 - 4 × LD50 | 100 | 1.20 | 90.8 |
|  | MDA-231 - 150% LD50 (1^st^ Exposure) | 93.37 | 1.22 | 17.9 |
|  | MDA-231 - 150% LD50 (2^nd^ Exposure) | 90.77 | 1.34 | 22.2 |
|  | MDA-231 - 150% LD50 (3^rd^ Exposure) | 99.53 | 1.07 | 28.8 |
|  | MDA-231 - 150% LD50 (4^th^ Exposure) | 100 | 1.08 | 39.2 |
|  | MDA-231 - 150% LD50 (5^th^ Exposure) | 97.93 | 1 | 60.8 |
|  | MDA-231 - 10% (Gradual) | 97.08 | 1.33 | 22.6 |
|  | MDA-231 - 20% (Gradual) | 96.98 | 1.40 | 22.0 |
|  | MDA-231 - 50% (Gradual) | 100 | 1.13 | 27.4 |
|  | MDA-231 - 100% (Gradual) | 100 | 1.02 | 42.2 |
|  | MDA-231 - 150% (Gradual) | 100 | 1.37 | 105.9 |
|  | MDA468 - NT | 88.56 | 1 | 8.9 |
|  | MDA-468 - 150% LD50 (1^st^ Exposure) | 91.78 | 1.39 | 16.9 |
|  | MDA-468 - 150% LD50 (2^nd^ Exposure) | 97.10 | 1.20 | 17.3 |
|  | MDA-468 - 150% LD50 (3^rd^ Exposure) | 99.11 | 1 | 30.7 |
|  | MDA-468 - 150% LD50 (4^th^ Exposure) | 98.71 | 1 | 39.1 |
|  | MDA-468 - 150% LD50 (5^th^ Exposure) | 100 | 1.11 | 59.1 |
|  | MDA-468 - 10% (Gradual) | 90.36 | 1 | 14.6 |
|  | MDA-468 - 20% (Gradual) | 86.92 | 1.24 | 21.0 |
|  | MDA-468 - 50% (Gradual) | 91.90 | 1 | 30.6 |
|  | MDA-468 - 100% (Gradual) | 100 | 1 | 45.6 |
|  | MDA-468 - 150% (Gradual) | 100 | 1 | 60.3 |
|  | AU565 – NT | 97.44 | 1.60 | 11.2 |
|  | AU565 - 150% LD50 (1st Exposure) | 100 | 1.12 | 6.3 |
|  | AU565 - 150% LD50 (2^nd^ Exposure) | 100 | 1.03 | 7.8 |
|  | AU565 - 150% LD50 (3^rd^ Exposure) | 99.3 | 1.15 | 7.5 |
|  | AU565 - 150% LD50 (4^th^ Exposure) | 100 | 1.02 | 6.7 |
|  | AU565 - 10% (Gradual) | 92.31 | 1.77 | 12.4 |
|  | AU565 - 20% (Gradual) | 96.48 | 1.45 | 14.1 |
|  | AU565 - 50% (Gradual) | 100 | 1.18 | 9.4 |
