# Supplementary material for: Heterogeneity and Plasticity of Human Breast Cancer Cells in Response to Molecularly-Targeted Drugs
Source: Front Oncol. 2019 Oct 15;9:1070. doi: 10.3389/fonc.2019.01070 (PMC6803545; doi:10.3389/fonc.2019.01070)

Supplementary Figure 1A. Scatterplots for protein expression profile - Ruxolitinib

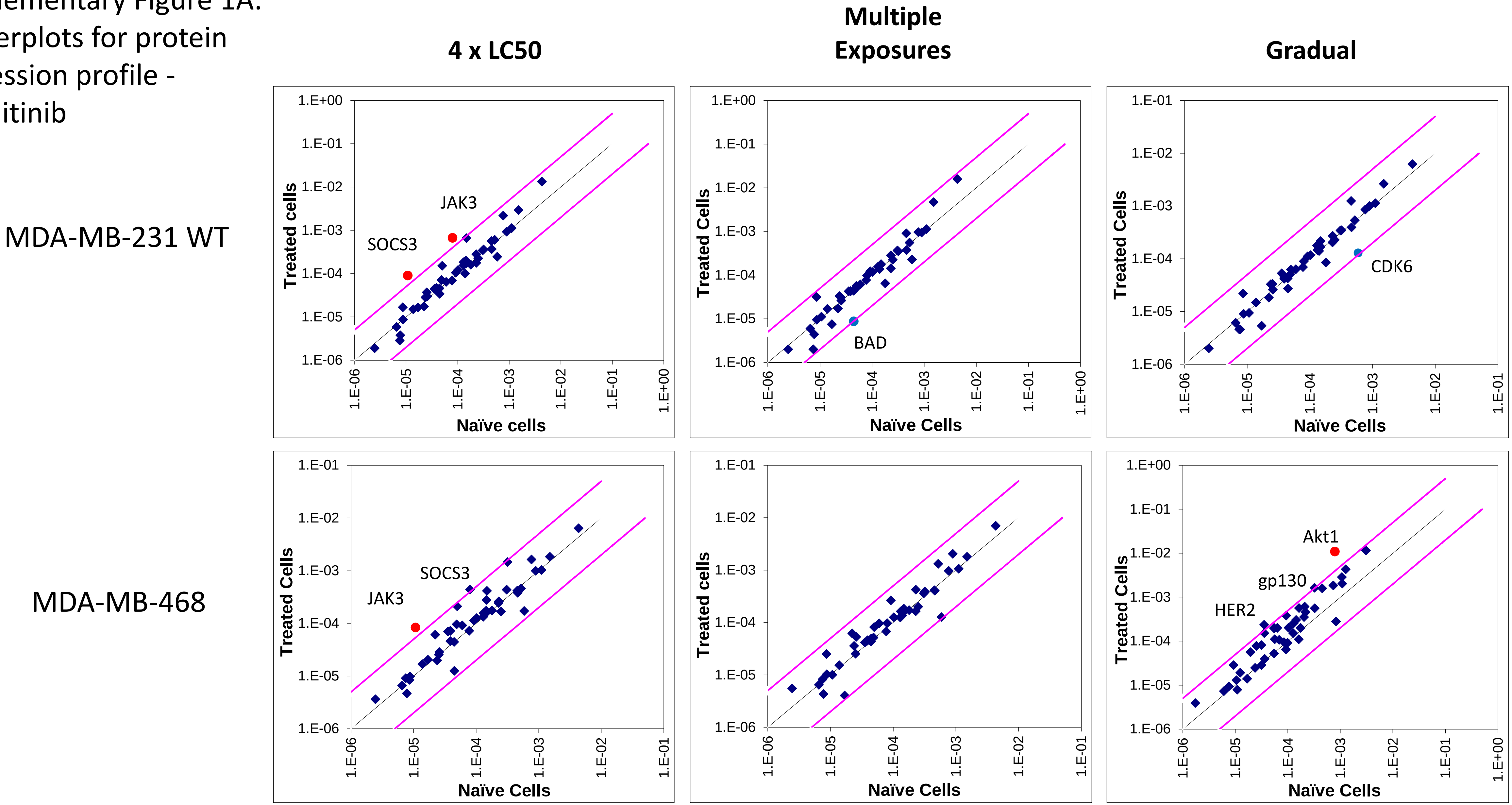

Supplementary Figure 1B. Scatterplots for protein expression profile - Everolimus

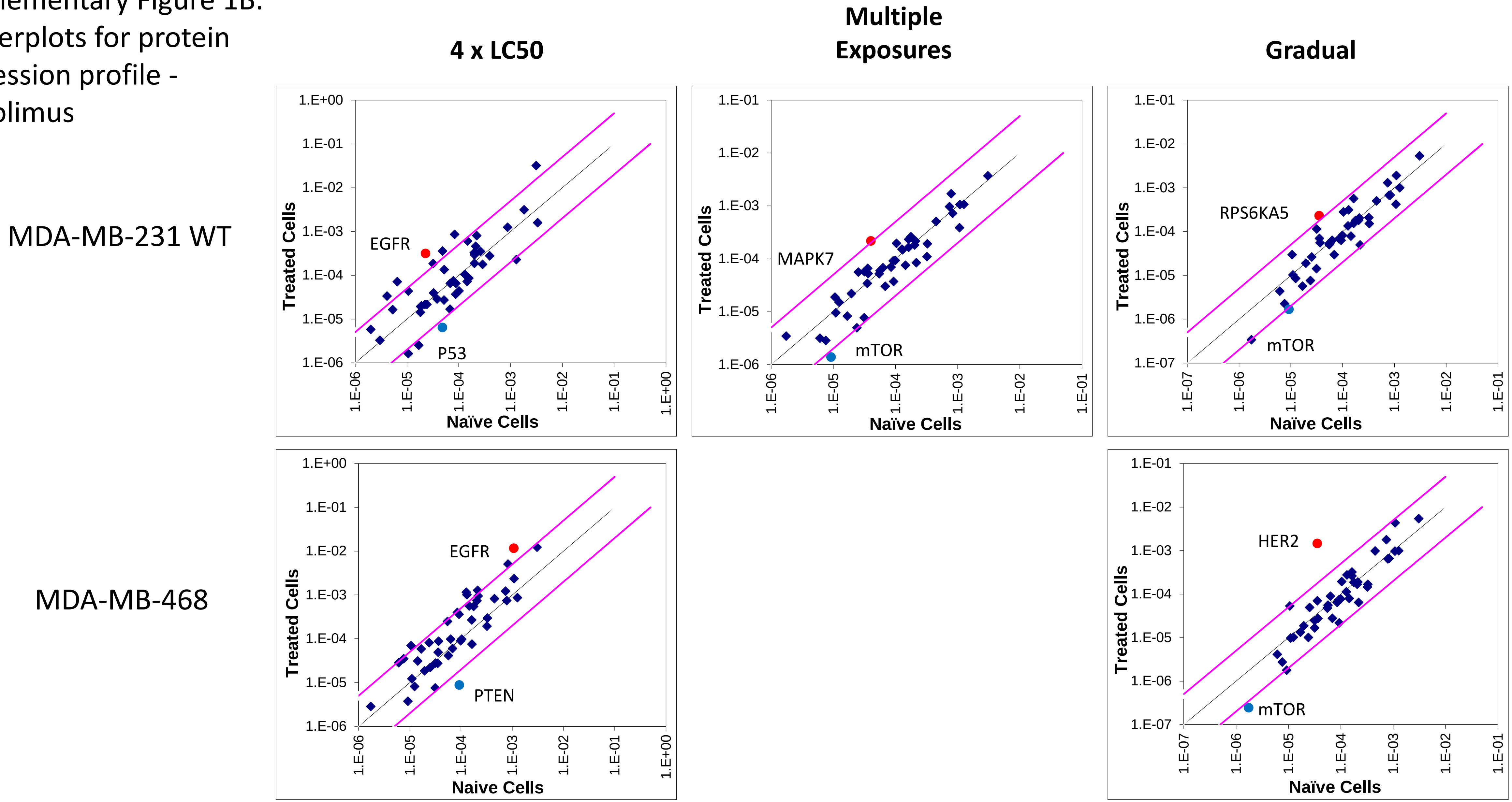

Supplementary Figure 1C. Scatterplots for protein expression profile - Erlotinib

	4 x LC50	Multiple Exposures	Gradual
MDA-MB-231 WT			
MDA-MB-468			

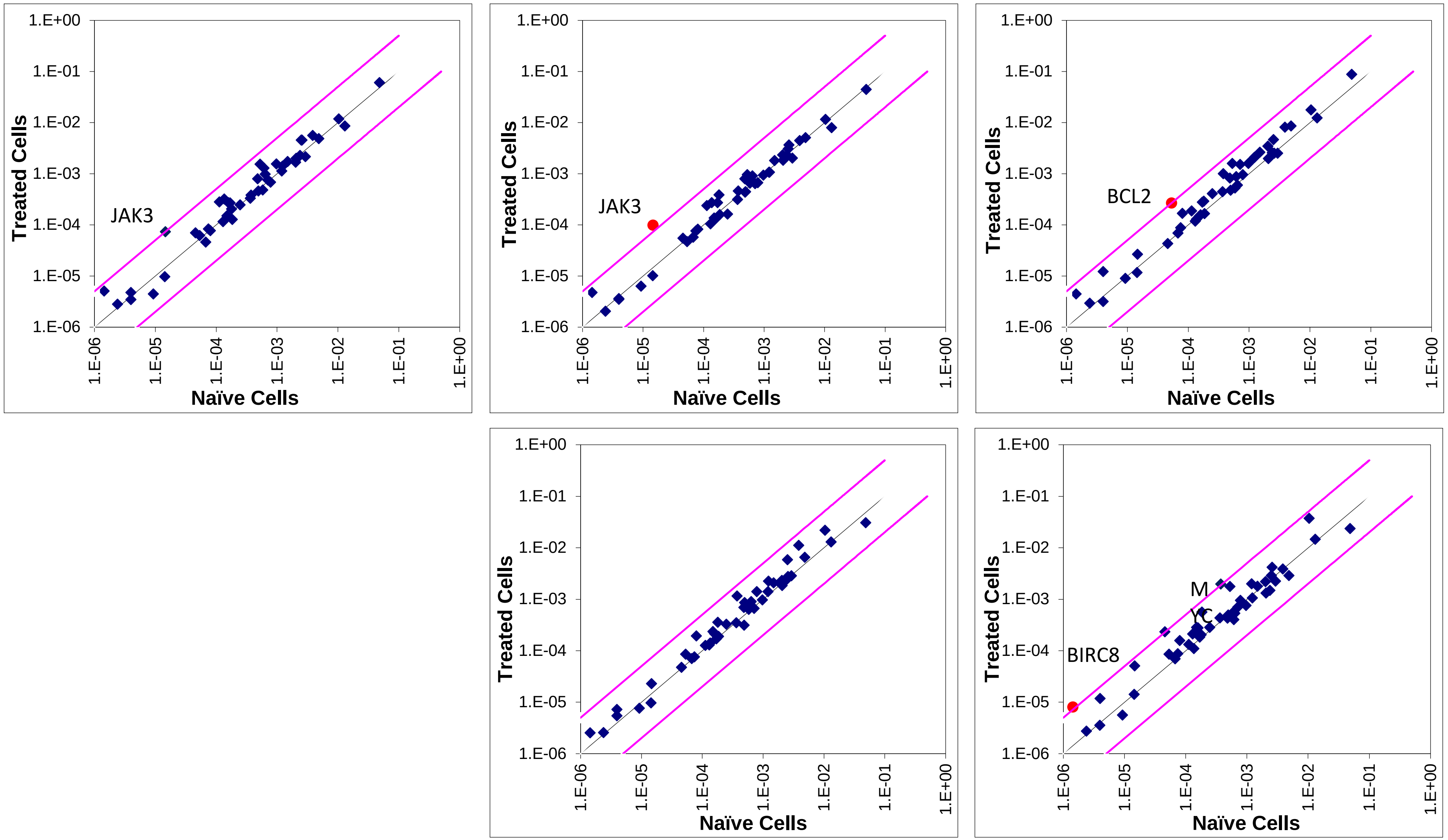

Supplement: Supplementary file 5 [file Image_1.pdf]
